# Suboptimal Weight Loss and Weight Regain after Gastric Bypass Surgery—Postoperative Status of Energy Intake, Eating Behavior, Physical Activity, and Psychometrics

**DOI:** 10.1007/s11695-016-2475-7

**Published:** 2016-12-02

**Authors:** Tina Amundsen, Magnus Strømmen, Catia Martins

**Affiliations:** 1 Centre for Obesity Research (ObeCe), Department of Surgery, St. Olavs Hospital—Trondheim University Hospital, Prinsesse Kristinas gate 3, 7030 Trondheim, Norway; 2 Obesity Research Group, Department of Cancer Research and Molecular Medicine, Faculty of Medicine, Norwegian University of Science and Technology, Trondheim, Norway; 3 Department of Neuroscience, Faculty of Medicine, Norwegian University of Science and Technology, Trondheim, Norway

**Keywords:** Morbid obesity, Gastric bypass, Suboptimal weight loss, Weight regain, Energy intake, Eating behavior, Physical activity

## Abstract

**Background:**

Suboptimal weight loss (SWL) and weight regain (WR) after gastric bypass surgery (GB) remains poorly understood.

**Objectives:**

This study aims to compare GB patients experiencing SWL or significant WR (SigWR) with successful controls, regarding postoperative food intake, eating behavior, physical activity (PA), and psychometrics.

**Methods:**

Forty-nine patients with >1 year post-surgery were classified as either experiencing SWL (excess body weight loss, EWL, <50%, *n* = 22) or SigWR (total weight regain ≥15%, *n* = 38), with respective control groups. Energy intake (EI) was measured with a Food Frequency Questionnaire, eating behavior using the Dutch Eating Behavior Questionnaire and the Three-Factor Eating Questionnaire, and PA using both SenseWear Armbands and the International Physical Activity Questionnaire. Eating disorders, depression, and quality of life (QoL) were measured using the Eating Disorder Examination Questionnaire, Beck Depression Inventory II, and Impact of Weight on Quality of Life, respectively.

**Results:**

EI, macronutrient distribution, and meal frequency were similar among groups. However, disinhibited eating behavior score was higher, while most subcategories from IWQOL were significantly lower in both SWL and SigWR groups compared with their respective controls. PA was significantly lower in the SWL and SigWR groups compared with the respective controls. There were no differences between groups regarding depression.

**Conclusions:**

Lower PA levels, disordered eating behavior and lower QoL are associated with unsuccessful weigh loss outcome after GB surgery. Longitudinal studies are needed to clarify the potential causal relationship between the previously described variables and SWL/SigWR after GB.

## Introduction

Gastric bypass (GB) is a common bariatric procedure for patients with severe obesity (BMI ≥40 kg/m^2^ or BMI ≥35 kg/m^2^ with comorbidities) [1]. The long-term effects of GB are well documented, both in terms of weight loss, improvement or resolution of obesity-related comorbidities, and risk factors [2], as well as improvement in quality of life (QoL) [3]. The exact mechanisms mediating the success of GB are still unknown, but changes in appetite hormones, bile acids, and microbiota have all been suggested [4].

Unfortunately, a subset of patients experience suboptimal weight loss (SWL), defined as either ≤50 or ≤40% excess body weight loss (EBWL) after GB surgery [5–8]. The prevalence of SWL increases over time. Approximately 20% of patients experience SWL 1 year after surgery [7, 8], while this proportion can reach as much as 33% at 10 years of follow-up [9]. In addition, weight regain (WR) has been reported in approximately 50% of GB patients within 2 years after surgery [10], with 15% gaining ≥15% weight from nadir [11].

Few studies have tried to identify factors responsible for SWL or WR after GB [5, 6, 12, 13]. Preoperative (greater initial BMI, male gender, and diabetes mellitus type 2 [6]) and postoperative factors (low dietary adherence [12] and high soda consumption [13]) have previously been associated with SWL or WR after GB. Overconsumption of energy-dense food postoperatively has also been associated with WR [14]. Eating behavior (including restrained, disinhibited, external, and emotional eating, and hunger perception) have shown to improve after GB [15, 16], and some studies suggest that preoperative scores may predict weight loss outcomes [12, 17]. Additionally, health-related QoL improves in most patients after bariatric surgery, but poor weight loss outcome, along with postoperative depression, can influence QoL negatively [18, 19].

More research is, therefore, required to improve our understanding of SWL and WR after GB, so that preventive strategies can be developed.

The primary aim of this study was to compare GB patients experiencing SWL or significant WR (SigWR) with successful controls, regarding postoperative food intake, eating behavior, and physical activity (PA). The secondary aim was to determine if an association exists between SWL/WR and QoL, depressive symptoms, and disordered eating.

## Subjects and Methods

### Study Design

This was a case-control study in which adult patients with SWL or significant WR (SigWR) after GB were compared with respective controls achieving an acceptable weight loss (AWL) and non-significant weight regain (NWR).

### Subjects

Participants were GB patients, who had undergone surgery a minimum of 1 year prior to this study. Invitation letters were sent to patients who complied to follow-up meetings at the Obesity Clinic at St. Olavs Hospital (Trondheim, Norway).

Exclusion criteria included pregnancy, enrollment in another obesity treatment, previous bariatric surgery other than GB, previous revisional bariatric surgery, known past or ongoing substance abuse, history of severe psychological disorder, severe eating disorder, and current medication known to affect body weight.

This study was conducted according to the guidelines laid down in the Declaration of Helsinki and was approved by the Ethics Committee (2012/1884, Central-Norway). Written informed consent was obtained from all participants before enrollment.

### Detailed Protocol

Participants were invited to St. Olavs Hospital for a screening interview, including anthropometric measurements and various questionnaires (all self-administered). Measurement of PA levels was, in addition to a questionnaire, also assessed with armbands. Participants were classified as either experiencing SWL (excess body weight loss (EBWL) < 50%, *n* = 21) or AWL (*n* = 27) (control group), and SigWR (total weight regain (TWR) > 15% *n* = 38) or NWR (TWR ≤ 15%, *n* = 11) (control group). A detailed description of how %EBWL and %TWR were calculated can be found below. The four groups were overlapping, meaning that participants could serve as a case in one group and a control in another group.

### Variables Collected

#### Anthropometrics

Height was measured without shoes, using a stadiometer (Seca, GmbH & Co., Germany) to the nearest 0.5 cm. Weight was measured to the nearest 0.1 kg, with light clothing and without shoes, using a digital scale (Seca, GmbH & Co., Germany).

Information on preoperative weight (operation day) was gathered from the participants’ hospital records. Lowest achieved weight after surgery was self-reported, usually recorded at a follow-up meeting 6 months to 2 years postoperatively.

The percentage of EBWL was calculated by dividing weight loss (kg) by excess weight [(preoperative weight − ideal weight) × 100]. Ideal weight was defined as a weight corresponding to a BMI of 25 kg/m^2^. Total weight regain (TWR) in percentage was calculated as (100/(preoperative weight − nadir weight)) × weight regain (kg). Total WL was calculated as preoperative weight − current weight, while actual WL as preoperative weight − nadir weight.

### Energy Intake and Eating Behavior

A 23-item Food Frequency Questionnaire (FFQ), validated on the Norwegian population [20], was used to collect data on total energy intake (EI), energy percentage (%E) provided by each macronutrient, intake of various food groups, and meal frequency. Participants were asked to have the last year in mind when reporting food intake. The FFQs were analyzed by the Medical Faculty at the University of Oslo, Norway.

Two questionnaires were used to measure eating behavior: the Three-Factor Eating Questionnaire (TFEQ) and the Dutch Eating Behavior Questionnaire (DEBQ). The TFEQ measures three different dimensions: restraint, disinhibition, and hunger [21]. The DEBQ also has three dimensions: emotional, external, and restrained eating [22].

#### Physical Activity

PA levels were measured with armbands (SenseWear Armband; BodyMedia, Pittsburgh, USA) and estimated by the International Physical Activity Questionnaire (IPAQ, Norwegian version) [23]. Participants were asked to use the armbands for 7 days. To be included in the analyses, each participant needed a minimum of 6 days of armband data, including two weekend days. One valid day was equal to 1368 min of data (95% of a 24-h period). The following variables were analyzed: average metabolic equivalent (MET); number of steps; time spent on sedentary, light, moderate, and vigorous activity; and total physical activity duration (min/day). Sedentary time was assumed as <1.5 MET, light as 1.5–2.9 METs, and moderate and vigorous activity as a MET between 3.0–5.9 and 6.0–8.7, respectively [24–27].

#### Psychometrics

Eating disorders were assessed by the Eating Disorder Examination (EDE)-Q 6.0 (Norwegian version) questionnaire [28]. EDE-Q has four subscales: dietary restraint, eating concern, weight concern, and shape concern, including a global score which is the average of the subscales. A global score of 4.0 has been suggested, but not validated, as a cut-off for disordered eating behavior [29].

Depression was assessed with the Beck Depression Inventory II (BDI-II, Norwegian version) [30]. The score ranges from 0 to 63, and the severity of depression is categorized in four groups (0–13 minimal, 14–19 mild, 20–28 moderate, and 29–63 severe depression).

QoL was assessed using Impact of Weight on Quality of Life-Lite (IWQOL-Lite, Norwegian version) [31].

### Statistical Analysis

Statistical analysis was carried out using SPSS 22.0 (SPSS Inc., Chicago, IL, USA). Data is reported as mean ± standard deviation (SD) for normally distributed variables and as median and interquartile range (IQR) for non-normally distributed variables. Statistical significance was assumed at *p* <0.05. Differences between groups were assessed by an independent samples *t* test for normally distributed continuous variables and Mann-Whitney *U* test for the non-normally distributed continuous variables (variables from FFQ, IWQOL-Lite, armbands, and IPAQ). Chi-square, along with Fisher’s exact test, was used for categorical variables.

The Benjamini-Hochberg method, which controls for the false discovery rate [32], was used to adjust for multiple comparisons. *P* values for group comparisons are shown both as unadjusted and adjusted.

## Results

### Subject Characteristics

A total of 51 participants met for the initial interview. Of those, two participants withdrew for unknown reasons, giving a total of 49 participants. The subject characteristics are displayed in Table 1.

**Table 1.** Characteristics of each group

Characteristics	SigWR (*n* = 36)	NWR (*n* = 11)	*p* value	SWL (*n* = 22)	AWL (*n* = 27)	*p* value
Female sex *n* (%)	31 (81.6)	11 (100)	0.124 (0.232)	18 (81.8)	24 (88.9)	0.482 (0.657)
Age (years)	45.5 ± 7.2	51.5 ± 7.5	0.02 (0.064)	46.5 ± 6.9	47.1 ± 8.2	0.815 (0.937)
Postoperative follow-up (months)	59.6 ± 26.0	53.9 ± 20.4	0.503 (0.663)	63.4 ± 27.6	54.2 ± 21.9	0.2 (0.358)
Preoperative BMI (kg/m^2^)	45.1 ± 6.1	44.7 ± 7.6	0.877 (0.937)	46.2 ± 6.6	44.0 ± 6.3	0.227 (0.385)
Actual WL (pre-nadir weight) %	56.6 ± 17.2	53.9 ± 17.8	0.233 (0.385)	59.1 ± 17.6	53.5 ± 16.7	0.006 (0.027)
Total WL (pre-current weight) %	18.8 ± 10.0	36.3 ± 6.4	<0.001 (0.002)	14.7 ± 8.7	28.8 ± 10.1	<0.001 (<0.001)
TWR %	43.7 ± 30.6	6.8 ± 6.0	<0.001 (<0.001)	56.3 ± 34.3	18.4 ± 13.0	<0.001 (<0.001)
EBWL %	44.8 ± 22.7	86.1 ± 22.5	<0.001 (<0.001)	29.8 ± 15.6	73.8 ± 19.7	<0.001 (<0.001)

Data presented as mean ± SD. *P* values are shown as unadjusted (adjusted for multiple comparisons)

*SigWR* significant weight regain, *NWR* non-significant weight regain, *SWL* suboptimal weight loss, *AWL* acceptable weight loss, *WL* weight loss, *TWR* total weight regain, *EBWL* excess body weight loss

The total sample consisted mainly of women (82%), with an average age of 46 years, preoperative BMI of 44.1 kg/m^2^, and postoperative follow-up time of 5 years. There were no significant differences in preoperative data or postoperative follow-up between any of the groups. TWR% was significantly higher in the SWL and SigWR groups, while total WL and %EBWL were significantly lower in the SWL and SigWR groups. No significant difference between any of the groups was found regarding actual WL (Table 1).

### Food Intake

Table 2 displays EI and %E from each macronutrient derived from the FFQ. No significant differences between none of the four groups were observed for EI or %E coming from carbohydrates, protein, and fat. The number of meals per day, breakfast frequency, snacking frequency, and intake of different food groups (data not shown) were also similar across all groups.

**Table 2.** Energy intake and %E from macronutrients (FFQ)

%E macronutrients	SigWR (*n* = 36)	NWR (*n* = 11)	*p* value	SWL (*n* = 21)	AWL (*n* = 26)	*p* value
Energy intake (kcal)	1910.0 (620.2)	1670.0 (582.0)	0.364 (0.523)	1879.0 (1129.0)	1849.0 (1104.0)	0.915 (0.937)
%E of protein	17.1 (5.9)	16.8 (4.7)	0.268 (0.427)	16.7 (2.3)	17.4 (4.4)	0.571 (0.722)
%E of carbohydrates	41.8 (8.8)	43.4 (11.8)	0.853 (0.937)	42.2 (6.5)	41.1 (9.1)	0.716 (0.867)
%E of added sugar	6.0 (6.3)	4.9 (2.8)	0.911 (0.937)	7.1 (6.0)	5.1 (5.5)	0.831 (0.937)
%E of fat	36.6 (10.3)	36.5 (7.8)	0.611 (0.750)	37.1 (10.6)	36.3 (8.7)	0.585 (0.729)
%E of fiber	2.3 (1.0)	2.5 (0.8)	0.931 (0.942)	2.3 (0.8)	2.4 (1.0)	0.915 (0.937)
%E of alcohol	0.3 (2.0)	2.0 (7.8)	0.057 (0.137)	0.0 (0.6)	0.9 (3.0)	0.015 (0.052)

Data presented as median (interquartile range). *P* values are shown as unadjusted (adjusted for multiple comparisons)

*SigWR* significant weight regain, *NWR* non-significant weight regain, *SWL* suboptimal weight loss, *AWL* acceptable weight loss, *%E* energy percentage

### Eating Behavior

Scores derived from the TFEQ and DEBQ are outlined in Table 3. There was a tendency for the disinhibition score from the TFEQ to be higher in SWL and SigWR groups compared to their respective controls. No other dimensions reached statistical significance.

**Table 3.** Eating behavior (TFEQ and DEBQ)

Eating behavior	SigWR (*n* = 38)	NWR (*n* = 11)	*p* value	SWL (*n* = 22)	AWL (*n* = 27)	*p* value
TFEQ						
Restraint	10.8 ± 3.9	10.5 ± 5.1	0.853 (0.937)	9.9 ± 3.9	11.4 ± 4.3	0.233 (0.385)
Disinhibition	7.9 ± 3.3	4.5 ± 4.0	0.02 (0.064)	8.1 ± 2.9	5.6 ± 3.7	0.015 (0.052)
Hunger	4.0 ± 2.4	3.9 ± 2.7	0.844 (0.937)	4.6 ± 2.5	3.5 ± 2.3	0.13 (0.238)
DEBQ						
Emotional	2.6 ± 1.0	2.3 ± 0.8	0.392 (0.562)	2.7 ± 0.8	2.5 ± 1.1	0.399 (0.563)
External	2.8 ± 5.7	2.4 ± 0.3	0.069 0.145	2.9 ± 0.5	2.6 ± 0.5	0.062 (0.137)
Restraint	2.9 ± 0.5	2.5 ± 0.7	0.05 0.126	2.9 ± 0.5	2.7 ± 0.6	0.517 (0.663)

Data presented as mean ± SD. *P* values are shown as unadjusted (adjusted for multiple comparisons)

*SigWR* significant weight regain, *NWR* non-significant weight regain, *SWL* suboptimal weight loss, *AWL* acceptable weight loss, *TFEQ* Three-Factor Eating Questionnaire, *DEBQ* Dutch Eating Behavior Questionnaire

### Physical Activity

Table 4 displays self-reported (IPAQ) and objectively measured (SenseWear Armbands) PA levels. Time spent on walking and total physical activity duration (IPAQ) were significantly lower in the SWL group. Regarding data derived from armbands, daily average MET, time spent on light PA, and total PA duration were significantly lower in the SWL and SigWR groups.

**Table 4.** Physical activity levels from IPAQ and armbands

Physical activity	SigWR (*n* = 38)	NWR (*n* = 11)	*p* value	SWL (*n* = 22)	AWL (*n* = 27)	*p* value
IPAQ (min/week)						
Walking	495.0 (2054.2)	792.0 (3799.1)	0.095 (0.186)	198.0 (858.0)	742.5 (1930.5)	0.005 (0.024)
Moderate activity	0.0 (480.0)	0.0 (840.0)	0.885 (0.937)	0.0 (240.0)	120.0 (480.0)	0.321 (0.493)
Vigorous activity	0.0 (690.0)	0.0 (1680.0)	0.383 (0.558)	0.0 (60.0)	0.0 (960.0)	0.209 (0.3687)
Total PA	862.5 (3048.3)	2748.0 (2560.5)	0.043 (0.112)	334.5 (1488.0)	2232.0 (3415.5)	<0.001 (0.005)
SenseWear armbands	SigWR (*n* = 25)	NWR (*n* = 9)		SWL (*n* = 14)	AWL (*n* = 20)	
Daily average MET	1.1 (0.2)	1.4 (0.3)	0.004 (0.020)	1.0 (0.3)	1.2 (0.2)	<0.001 (0.005)
Sedentary activity (min/day)	1199.0 (112.0)	1118.0 (205.5)	0.03 (0.086)	1207.0 (149.0)	1136.0 (140.0)	0.061 (0.137)
Light activity (min/day)	153.0 (80.2)	247.0 (71.2)	0.004 (0.020)	145.0 (75.0)	223.5 (105.0)	0.015 (0.052)
Moderate activity (min/day)	41.0 (39.5)	81.0 (100.0)	0.06 (0.137)	35.0 (46.0)	61.5 (49.0)	0.056 (0.137)
Vigorous activity (min/day)	0.0 (1.2)	0.0 (5.0)	0.489 (0.657)	0.0 (1.0)	0.0 (4.0)	0.743 (0.875)
Total PA duration (min/day)	195.0 (112.0)	314.0 (152.7)	0.003 (0.018)	181.0 (122.0)	276.0 (114.0)	0.0007 (0.031)
Number of steps/day	4919.0 (3978.7)	5686.0 (5539.3)	0.489 (0.657)	4777.5 (3098.0)	5688.0 (3361.0)	0.245 (0.398)

Data presented as median (interquartile range). *P* values are shown as unadjusted (adjusted for multiple comparisons)

*SigWR* significant weight regain, *NWR* non-significant weight regain, *SWL* suboptimal weight loss, *AWL* acceptable weight loss, *IPAQ* International Physical Activity Questionnaire, *MET* total average metabolic equivalent of task. Sedentary activity: <1.5 MET (minutes). Light activity: 1.5–3.0 MET (minutes). Moderate activity: 3.1–5.9 MET (minutes). Vigorous activity: 6.0–8.7 MET (minutes). Physical activity duration: >1.5 MET (minutes)

### Psychometrics

Table 5 outlines psychometrics derived from the IWQOL-Lite, EDE-Q, and BDI-II. Median score for most subcategories in IWQOL-Lite were significantly lower in SWL and SigWR groups compared with respective controls.

**Table 5.** Psychometrics (IWQOL, EDE-Q, and BDI-II)

Questionnaire	SigWR (*n* = 38)	NWR (*n* = 11)	*p* value	SWL (*n* = 21)	AWL (*n* = 27)	*p* value
IWQOL-Lite						
Physical functioning	82.9 (26.7)	95.4 (7.3)	0.004 (0.020)	79.5 (30.6)	88.6 (13.6)	0.002 (0.014)
Self-esteem	66.0 (56.2)	96.4 (14.0)	0.014 (0.052)	58.9 (41.0)	85.7 (25.0)	<0.001 (0.005)
Sexual life	87.5 (51.5)	100 (0.0)	0.065 (0.140)	75.0 (46.8)	100.0 (18.7)	0.013 (0.05)
Public distress	85.0 (35.0)	100 (0.0)	0.03 (0.086)	82.5 (37.5)	100.0 (15.0)	<0.001 (0.008)
Work	100 (18.7)	100 (0.0)	0.366 (0.543)	93.7 (31.2)	100.0 (0.0)	0.037 (0.099)
EDE-Q				SWL (*n* = 21)	AWL (*n* = 26)	
Restraint	1.3 ± 0.9	1.1 ± 1.3	0.509 (0.663)	1.1 ± 0.7	1.4 ± 1.2	0.29 (0.453)
Eating concern	0.9 ± 1.0	0.8 ± 0.9	0.732 (0.874)	0.9 ± 1.0	0.8 ± 1.1	0.883 (0.937)
Shape concern	2.9 ± 1.3	2.0 ± 1.8	0.074 (0.152)	3.4 ± 1.0	2.2 ± 1.6	0.008 (0.033)
Weight concern	2.6 ± 0.9	1.3 ± 1.7		2.9 ± 0.7	1.8 ± 1.4	0.001 (0.008)
Global score	1.9 ± 0.8	1.3 ± 1.2	0.074 (0.152)	2.0 ± 0.6	1.5 ± 1.1	0.111 (0.212)
BDI-II	SWR (*n* = 38)	NWR (*n* = 11)		SWL (*n* = 22)	AWL (*n* = 27)	
Total score	9.0 ± 7.9	9.2 ± 10.3	0.946 (0.946)	11.9 ± 8.6	6.7 ± 7.5	0.029 (0.086)
Minimal depression, *n* (%)	30 (78.9)	9 (81.8)		15 (68.2)	24 (88.9)	
Mild depression, *n* (%)	3 (7.9)	0 (0.0)		2 (9.1)	1 (3.7)	
Moderate depression, *n* (%)	4 (9.1)	1 (10.5)		4 (18.2)	1 (3.7)	
Severe depression, *n* (%)	1 (2.6)	1 (9.1)		1 (4.5)	1 (3.7)	

Data for EDE-Q and BDI-II total score presented as mean ± SD. *P* values are shown as unadjusted (adjusted for multiple comparisons). Depression categories presented as *n* (% within each group). Data for IWQOL presented as median (interquartile range)

*SigWR* significant weight regain, *NWR* non-significant weight regain, *SWL* suboptimal weight loss, *AWL* acceptable weight loss

Regarding eating disorders, the only significant difference found was for weight and shape concern, which were higher in the SWL group. No significant difference between groups was found on the BDI-II total score (or the percentage of participants in each depression group).

## Discussion

In the present study, participants experiencing either SWL or SigWR were less physically active and presented with higher disinhibition and weight and shape concern scores, compared to participants with AWL or NWR. Moreover, they also presented with an overall lower QoL. In accordance with our expectations, several self-reported and objectively measured PA variables were found to be significantly lower in the SWL and SigWR groups. This is in line with several other studies showing that PA is important in weight maintenance and prevention of weight regain after GB [33–35]. Participants who engage in >150 min of moderate to vigorous PA were previously found to have a greater %EBWL than those who were less physically active 2–5 years after GB [36]. Self-reported moderate and total PA have also been reported to correlate positively with %EBWL [37].

The American College of Sports Medicine (ACSM) recommends >250 min per week of moderate PA to prevent weight regain [38]. In the present study, the AWL group had an average of 431 min of moderate PA per week (61.5 min/day), while the NWR group had 567 min per week (81.0 min/day), which is well above the ACSM’s recommendation. Time spent on moderate activity in the SWL and SigWR groups was almost half of that seen in the respective control groups, but in accordance with the recommendations (on average 266 min/week). These results suggest that the present PA recommendations proposed by the ACSM to prevent weight regain may not be sufficient after GB.

Contrary to our expectations, no significant differences in total EI, macronutrient distribution, intake of different food groups, and meal/breakfast frequency were found between any of the groups. It needs to be emphasized, nevertheless, that the SigWR group had an energy intake which was 14.4% (240 kcal) higher than the NWR group and larger studies could potentially show significant differences between groups. Weight regain after GB has previously been associated with poor diet quality (higher caloric intake, sweets, snacks, and fatty foods) [33], while a lower daily EI has been found to correlate with %EBWL [39, 40]. However, two other studies reported no correlation between EI and %EBWL after GB surgery [41, 42]. Given the larger postoperative BMI in the SWL and SigWR groups (assuming that participants were weight stable), a higher total EI would be expected in those groups. The fact that food intake was self-reported and may suffer from underreporting and the small sample size of the study may therefore have biased the results.

Disinhibition (TFEQ) score was found to be significantly higher in both the SWL and SigWR groups. Previous studies have shown inconclusive results and, unlike the present study, tend to present only preoperative scores [12, 17, 43]. Lower disinhibition and hunger scores (TFEQ) have been reported 1 year after adjustable gastric banding or vertical banded gastroplasty on those successful in weight loss (versus unsuccessful) [44]. To our knowledge, only one study has examined DEBQ and weight loss after GB [43]. Opposite to our results, the study found an inverse relationship between weight loss and emotional and external eating, but no relationship with restrained eating [43].

Regarding the EDE-Q, weight and shape concern were found to be significantly higher in both the SWL groups. However, all groups had average values below the cut-off for disordered eating. Still, the presence of a sub-clinical eating disorder cannot be fully excluded. Some studies have found that EDE-Q scores significantly improve after GB [45, 46]. Very few studies have examined the relationship between disordered eating and unsuccessful weight loss after GB. Hrabosky and colleagues (2006) reported no correlation between the degree of weight loss and changes in EDE-Q scores postoperatively [45]. Another study also found improvements in different aspects of eating symptomatology (using EDI-3), but reported no correlation between such symptoms and weight loss after GB [47].

We found no significant differences in depressive symptoms between groups, and the average total score was in the minimal depression category for all. Several studies have reported an improvement in depressive symptoms after various bariatric surgery procedures, regardless of weight loss [42, 48–50]. However, Faulconbridge and colleagues (2013) reported a positive correlation between improvement in depression scores (BDI-II) postoperatively and the degree of weight loss [51]. Another study found preoperative BDI score to be positively correlated with the amount of weight lost 1 year after GB surgery [52].

Consistent with our expectations, most dimensions of the IWQOL-Lite were significantly lower in the SWL and SigWR groups, suggesting that those unsuccessful after GB have a lower QoL. A previous study has reported similar QoL improvements in both successful and unsuccessful weight loss participants (<50% EBWL) 8 years after GB [42]. A significant improvement in QoL was also reported in another study, using the SF-36 questionnaire (a generic QoL-assessment tool), with greater improvement seen in patients with the largest weight reduction [53].

It is important to note that our unsuccessful participants, regarding weight loss outcome after GB, regardless of the definition used (SWL based on EWL% or SigWR), presented with no significant difference in actual WL (given as preoperative − nadir weight) compared with those successful (AWL and NWR). However, both the SWL and SigWR groups had a significantly lower total WL (given as preoperative − current weight) and a significantly higher TWR%. Thus, successful weight loss outcome after GB surgery was due to the degree of weight regain and not actual weight loss. Limitations in this study include its small sample size, which may underpower it statistically, its cross-sectional design and subsequently the lack of preoperative measurements, and the fact that the lowest achieved weight was self-reported. Moreover, changes in post-surgical anatomy, which can affect weigh regain, were not looked at. Despite these limitations, the current findings are significant and can help clinicians better manage their patients.

In conclusion, lower PA levels, disordered eating behavior, and lower QoL are associated with unsuccessful weight loss outcome after GB, defined either as SWL or SigWR. Future studies, with a longitudinal design and larger sample sizes, are needed to clarify the causal relationship between SWL and WR after GB and the previously described variables.
